# Different immunogens and *prime-boost* vaccination strategies affect the efficacy of recombinant candidate vaccines against pathogenic orthopoxviruses

**DOI:** 10.1186/s12985-024-02534-4

**Published:** 2024-11-07

**Authors:** Antonia Radaelli, Carlo Zanotto, Chiara Brambilla, Tommaso Adami, Francesca Paolini, Aldo Venuti, Adriana Manuka, Irsida Mehmeti, Carlo De Giuli Morghen

**Affiliations:** 1 Department of Medical Biotechnologies and Translational Medicine, University of Milan, via Vanvitelli 32, Milan, 20129 Italy; 2 Faculty of Pharmacy, Catholic University “Our Lady of Good Counsel”, Rr. Dritan Hoxha, 123, Tirana, Albania; 3 UOSD Tumor Immunology and Immunotherapy, HPV UNIT, IRCCS Regina Elena National Cancer Institute, via Chianesi, 53, Rome, 00144 Italy; 4 Laboratory of Molecular Virology and Recombinant Vaccine Development, Department of Medical Biotechnologies and Translational Medicine, University of Milan, Via Vanvitelli, 32, Milan, 20129 Italy

**Keywords:** *Prime-boost* immunization regimens, Recombinant vaccines against MPXV, Orthopoxvirus vaccines, Enhancement of the immune response

## Abstract

Although not as lethal as variola virus (VARV), the cause of smallpox, monkeypox virus (MPXV) represents a threat to public health, with important infection rates and mortality in several African countries and signs of spreading worldwide. MPXV may establish new reservoirs in non-endemic countries and can be considered a possible biological weapon. Human-to-human MPXV transmission is increasing with a growing susceptibility, coincident with the declining herd immunity against smallpox. The emerging threat of MPXV highlights the urgent need for protection from new zoonotic infections, as mankind is completely unprepared for encounters with new viruses. Preventive vaccination remains the most effective control against orthopoxviruses (OPXVs) such as MPXV and *prime-boost* vaccination strategies can significantly influence vaccine efficacy and enhance immune responses. Our study aimed at characterizing potential vaccine candidates against OPXV infections in a murine model using DNA, viral and protein recombinant vaccines using different *prime-boost* regimens. The experiments employed Vaccinia virus (VACV) A33, B5, L1, and A27 envelope proteins as immunogens for both *priming* and *boosting*. *Priming* was carried out using a mixture of four plasmids (4pVAXmix), and *boosts* employed fowlpox (FWPV) recombinants (4FPmix) and/or the purified recombinant proteins (4protmix), all of them expressing the same antigens. One or two doses of the same immunogens were tested and identical protocols were also compared for intranasal (i.n.) or intramuscular (i.m.) viral administration, before challenge with the highly pathogenic VACV VV_IHD−J_ strain. Our results show that a single dose of any combined immunogen elicited a very low antibody response. Protein mixtures administered twice boosted the humoral response of DNA immunizations by electroporation (e. p.), but did not protect from viral challenge. The antibody neutralizing titer was inversely correlated with animals’ weight loss, which was initially similar in all of the groups after the challenge, but was then reversed in mice that had been primed twice with the DNA recombinants and boosted twice with the FWPV recombinants.

## Highlights

Recombinant avipoxviruses as putative recombinant vaccines against MPXV.

Different *prime–boost* regimens to improve immunization against orthopoxviruses.

Mucosal and intramuscular immunization to enhance the humoral and neutralizing immune response.

## Introduction

Due to the successful eradication of smallpox worldwide, vaccination against Variola virus (VARV), the etiological agent of smallpox, which uses humans as exclusive hosts, was discontinued in 1980. Samples of VARV are officially stored only in two high-security BSL-4 laboratories (US CDC, Atlanta, and Russia State RCVB, Koltsovo, Novosibirsk). Despite these precautions, the accidental or deliberate release of VARV or other pathogenic orthopoxviruses (OPXVs) as biological weapons is still a major concern [1].

Although not as lethal as VARV, monkeypox virus (MPXV) represents a threat to public health [2] as it causes high levels of illness and death with a case-fatality rate of around 10% [3, 4]. Several periods of uninterrupted spread among humans have been reported [5] in the Democratic Republic of Congo (DRC), and, during the second half of 2013, MPXV cases increased 600-fold in the Bokungu Health Zone of the DRC [3, 6]. The MPXV global outbreak in 2022 spanned more than 100 countries, indicating a potential increase in human-to-human transmission [5–10], possibly influenced by multiple factors. Among these factors is the declining smallpox herd immunity, a trend observed for over 44 years. Protection from new zoonotic viruses is urgently needed [11–14] and, in the case of OPXVs such as MPXV, preventive vaccination remains the most effective form of control against viral infection.

The smallpox vaccine consisted of live preparation of vaccinia virus (VACV). More recently, attenuated avipoxviruses have been developed as novel vectors for the construction of recombinant vaccines against several human infectious diseases [15–17]. These vectors are naturally restricted to avian species for their replication, although they are permissive for entry in most mammalian cells [18, 19]. They do not cross-react immunologically with mammalian poxviruses, but they express transgenes correctly [20]. Recombinants can accept the insertion of up to 25 kbp of foreign gene sequences and can simultaneously express multiple genes [18]. Prophylactic clinical trials, mainly using canarypox (ALVAC) or fowlpox (FWPV) recombinants, showed a potential protective efficacy against HIV, malaria and tuberculosis [21, 22]. The RV144 Phase-III trial in Thailand [23], the immunization strategy of which included an HIV-1 *gag*, *protease* and *env*-expressing ALVAC vector, indicated that an avipox vaccine could protect against HIV-1 infection, albeit with limited efficacy. Sequential *prime-boost* immunizations with attenuated poxvirus vectors (FP9 and MVA), expressing six pre-erythrocytic malaria antigens from *Plasmodium falciparum*, were also evaluated [24] in a Phase I/IIa-challenge trial. The safety and immunogenicity of a new candidate tuberculosis avipox-based vaccine was also evaluated in a Phase-I clinical trial [25]. Because of the absence of cross-reactivity with VACV, avipoxviruses can also escape neutralization by vector-generated antibodies in smallpox-vaccine experienced humans [26] and they represent safe immunogens for most mammalian cells [27, 28]. Fowlpox has received approval for clinical use in humans (www.ClinicalTrials.gov Identifier: NCT00083603).

After vaccination, VACV neutralizing antibodies are mainly raised against the A33 and B5 proteins of the extracellular virions (EV) and against the L1 and A27 proteins of intracellular mature virions (MV), which are released after cell lysis [29–32]. These proteins have been shown to be protective in mice after intranasal (i.n.) VV_IHD−J_ challenge, and in monkeys after intravenous (i.v.) MPXV inoculation [33–36]. Thus, subunit vaccines that express the VACV A33, B5, L1, and A27 surface proteins have been designed and showed to be protective also in monkeys after intravenous MPXV inoculation. L1 protein expression was also improved by the construction of a FWPV-based immunogen, endowed with the signal sequence of tissue plasminogen activator (tPA). This sequence, added to the 5’ end of the *L1R* open reading frame, is able to increase the expression and immune efficacy of L1, which in its unmodified form is not efficiently transported to the cell surface [37, 38].

DNA vaccines are relatively thermostable, inexpensive, easily susceptible to genetic manipulations and they are safe also for immunocompromised individuals, as they do not contain live or inactivated pathogens [39]. Endogenous synthesis of the immunogens allows antigen presentation in association with class-I and class-II major histocompatibility complex molecules (MHC-I and MHC-II), thus activating both the humoral and cellular arms of the immune system. Thus, they have also been used as a *prime* to enhance the immunogenicity of poxvirus-based recombinants [37, 40-42].

The administration route can be an important determinant of the efficacy of a vaccine. It is known that most pathogens enter their host through mucosal sites, but most vaccines are administered by the subcutaneous (s.c.) or intramuscular (i.m.) route and only a few mucosal vaccines have been approved for human use. FWPV is an excellent mucosal delivery vector, compared to recombinant DNA or VACV [43–46] and its inter-human transmission may be favored by close contact [47]. The i.n. route can also be more practical than the i.m. route [48], and thus facilitate mass vaccination campaigns.

Here, we evaluated, in a surrogate animal model of MPXV infection, the preventive and protective activity of DNA genetic vaccines administered by in vivo s.c. inoculation and electroporation (e.p.), followed by i.n. or i.m. administration of FWPV recombinants or, alternatively, s.c. inoculation of recombinant proteins. *Priming* was always performed with a mix of four different recombinant pVAX DNA plasmids that express the VACV A33, B5, L1, and A27 envelope proteins (4pVAXmix). The mice were then boosted with FWPV recombinants that carried the same VACV genes (4FPmix) or with a mixture of purified recombinant A33, B5, L1, and A27 proteins (4protmix) to enhance the antibody response. Vaccinations were performed using either one or two shots of the immunogens to determine whether single or double administrations produced the same results. Two identical protocols were also compared for i.n. or i.m. viral immunization, alternatively. Only mice primed twice with 4pVAXmix and boosted twice, either i.n. or i.m., with 4FPmix, were protected after the challenge with the highly pathogenic VV_IHD−J_ virus. This response correlated with recovery from challenge-induced weight loss and a higher antibody neutralizing titer.

## Materials and methods

### Fowlpox recombinants and VACV IHD-J challenge virus

The 4 FWPV recombinants, FP_*tPA−L1R*_, FP_*A27L*_, FP_*A33R*_, and FP_*B5R*_, expressing the VACV L1, A27, A33, and B5 proteins, respectively, were generated by in vivo site-specific homologous recombination (IVR) between the DH gene of the wild-type FWPV virus (FPwt, 5 PFU/cell) and their respective recombination plasmids, essentially as described [28, 49, 50]. The FP_*tPA−L1R*_ recombinant was recently constructed [38] to replace FP_*L1R*_, the protein expression of which was very low. All of the recombinants were amplified in CEFs and purified on discontinuous sucrose gradients, as described previously [28]. The highly pathogenic IHD-J strain of VACV (VV_IHD−J_) was supplied by S. Dales (University of Western Ontario, London, Canada), and was used as the challenging virus (1 × 10^7^ PFU/mouse, i.e., 5-fold the LD_50_), with i.n. administration. VV_IHD−J_ was grown in Vero cells, then amplified, purified on a discontinuous sucrose density gradient, and titrated, as described previously [28].

### Immunization protocols

**Fig. 1:**
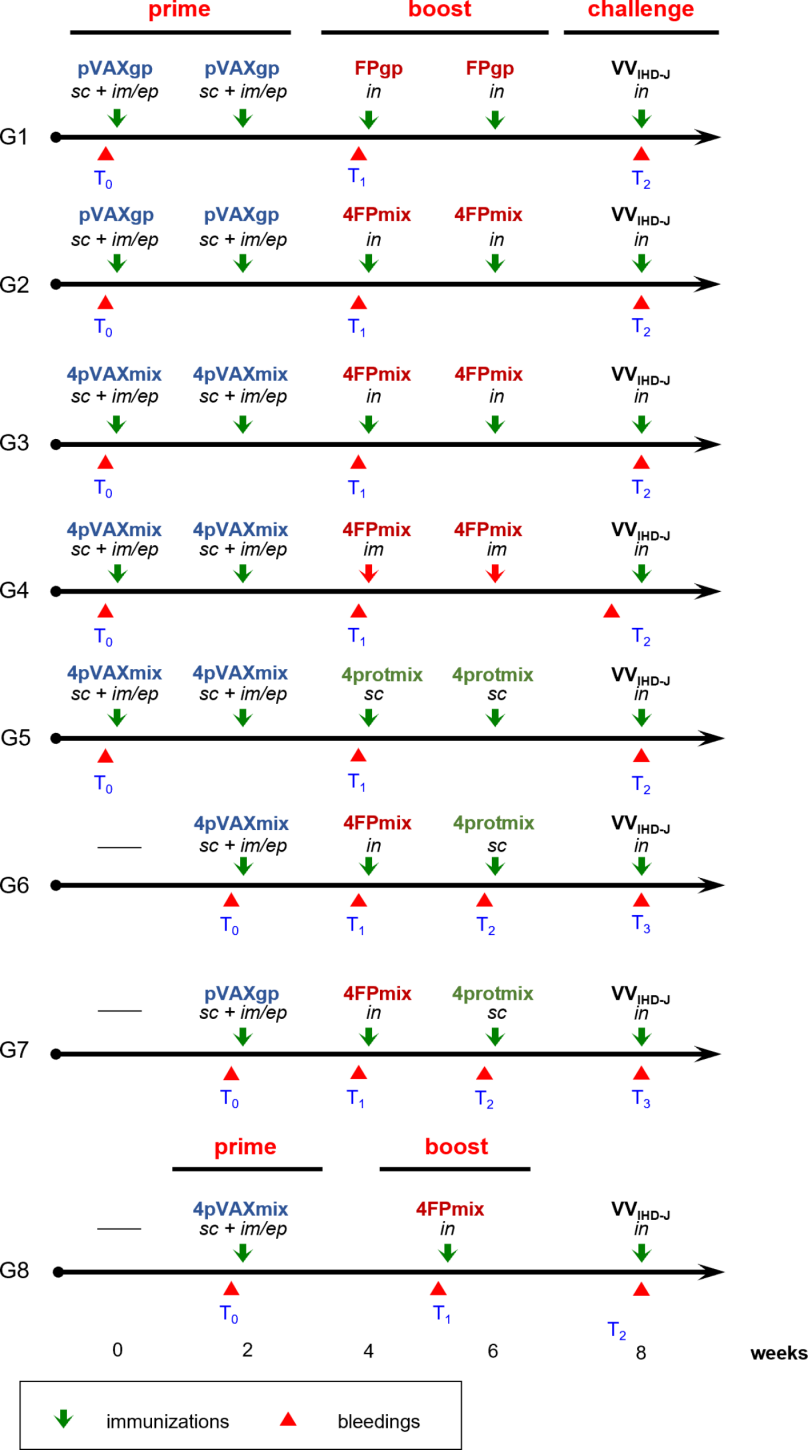
**Immunization protocols.** Eight different regimens (G1-G8) were followed using 7 mice per group. All of the immunogens expressed the VACV *L1R*, *A27L*, *A33R*, and *B5R* gene products and were administered twice (G1-G5) or once (G6-G8). DNA recombinants (4pVAXmix, in blue) were always used for *priming* and injected s.c. on the back (12 µg/mouse, 3 µg/recombinant) or i.m./e.p on the leg (48 µg/mouse, 12 µg /recombinant). Viral recombinants (4FPmix, in red) were used as *boosts* and administered i.n. (4 × 10^6^/mouse, 1 × 10^6^ PFU/recombinant) except for G4 where i.m. administration was performed. Protein recombinants (4protmix, in green) were used for the *boost* and administered s.c. (40 µg/mouse, 10 µg of each recombinant protein). pVAX*gp* and FP*gp* recombinants, containing HIV-1 *gag/pro* genes, were used as irrelevant immunogens. The VV_IHD−J_ challenge virus was administered i.n. at 1 × 10^7^ PFU/mouse (i.e. 5-fold the LD_50_). Blood samples were obtained from the mice before the first immunization (T0), before the *boosts* (T1, T2) and just before the VV_IHD−J_ challenge (T3)

Experiments were performed on eight groups of 8-week-old female BALB/c mice without oestrous cycle synchronization (Charles River Laboratories, Wilmington, MA), with seven mice/group. Before each immunization, the mice were anaesthetized by isofluorane (1.5 L/min) and the immunogens were administered as shown in Fig. 1. The same immunogens were administered once or twice both for *priming* and for *boosting* (Fig. 1). Specifically, group 1 (G1) received 2 doses of pVAX*gp* followed by 2 doses of FP*gp* recombinant; group 2 (G2), 2 doses of pVAX*gp* followed by 2 doses of 4FPmix recombinants; group 3 (G3), 2 doses of 4pVAXmix followed by 2 doses of 4FPmix recombinants; group 4 (G4), 2 doses of 4pVAXmix followed by 2 doses of 4FPmix recombinants; group 5 (G5), 2 doses of 4pVAXmix followed by 2 doses of 4 protmix; group 6 (G6), one dose of 4pVAXmix followed by one dose of 4FPmix recombinants and one dose of 4protmix; group 7 (G7), one dose of pVAX*gp* followed by one dose of 4pFPmix recombinants and one dose of 4protmix; group 8 (G8), one dose of 4pVAXmix followed by one dose of 4FPmix recombinants. *Priming* with the 4pVAXmix plasmid preparation was carried out by s.c. injection of 12 µg (i.e. 3 µg of each recombinant/mouse) on the animals’ backs, or by i.m. injection followed by e.p. of 48 µg (i.e. 12 µg of each recombinant/mouse) in the leg. The DNA dosage and the anatomical localization for e.p. delivery was previously determined and already utilized in previous studies [51, 52]. Electroporation (one 50-ms transcutaneous low-voltage electric pulse, amplitude, 100 V) was applied at the i.m. injection site of the leg with a tweezertrode connected to an electroporator apparatus (ECM830, BTX i45-168, Holliston, MA), using three orthogonal planes to cover the maximum area. The *boosts* were performed with FWPV recombinants and/or recombinant proteins. FWPV recombinants were administered i.n. or i.m. at 4 × 10^6^ PFU/mouse (1 × 10^6^ PFU of each recombinant). Recombinant proteins, a kind gift of Luca Vangelista (Nazarbayev University School of Medicine, Department of Biomedical Sciences, Astana, Kazakhstan) [53], were administered s.c. at 40 µg/mouse (10 µg of each protein). Both pVAX*gp* plasmid and FP*gp*, which contain the SIVmacM766 *gag/pro* gene, were used as irrelevant immunogens [37]. Each immunization was performed at two-week intervals. Two weeks after the last immunization, the mice were challenged i.n. with a lethal dose of VACV VV_IHD−J_.

### Blood sampling and monitoring

Blood samples were obtained from the sub-mandibular plexus before the first immunization (Fig. 1, T0), before each immunogen change (Fig. 1, T1, T2), and just before the challenge (Fig. 1, T2/T3). The challenge for the different immunization protocols was performed using 5-fold the LD_50_ (i.e., 1 × 10^7^ VV_IHD−J_ PFU/mouse), as previously determined [37]. The serum fraction was aliquoted and frozen at -80 °C. Animals were monitored during the whole treatment period for weight loss and signs of disease, and were provided with food and water *ad libitum* until euthanasia. Every effort was made to minimize their suffering, and they were euthanized based on the predetermined criterion of loss of > 25% body weight. Approval for this study protocol was granted by the Ethical Committee of the University of Milan. All of the mice were maintained according to the Italian National Guidelines and the EU Directive 2010/63/EU for animal experiments.

### Enzyme-linked immunosorbent assay (ELISA)

The mouse serum samples were tested for antibodies against the VACV-specific A33, A27, B5, and L1 proteins (BEI Resources, Manassas, VA). Proteins were plated as 100 ng of each protein/well in 96-well microtiter plates (MaxiSorp; Nunc, ThermoFisher Scientific, Roskilde, Denmark) in 0.05 M carbonate-bicarbonate buffer, pH 9.6 (15 mM Na_2_CO_3_, 35 mM NaHCO_3_, 0.2% NaN_3_), and incubated overnight at 4 °C. ELISAs were performed in triplicates, as described previously [17], using the pooled sera of each group of mice from T0, T1, T2, and T3 (see Fig. 1). Sera dilutions were 1:100 for A33 protein; 1:00 and 1:2000 for A27 protein;  1:100 and 1:500 for B5 protein; and 1:2000 and 1:10000 for L1 protein. The reactions were revealed using a 1:5000 dilution of goat anti-mouse horseradish-peroxidase-conjugated antibody (Fisher Scientific) and tetramethylbenzidine substrate (Sigma–Aldrich). The pre-immunization mouse sera (Fig. 1, T0) were used as negative controls. The absorbance of each well was read at 450 nm using a Microplate Reader 550 (Bio-Rad, Hercules, CA).

### Virus plaque reduction neutralization test (PRNT)

The neutralizing activities of the mice sera obtained before the challenge were determined by measuring the extent of in vitro inhibition of VACV infectivity. The assays were performed as previously described [37] by pre-incubation of an equal volume of VV_IHD−J_ with diluted heat-inactivated mouse serum in 48-well plates, for 1 h at 37 °C. The viral titer was adjusted to provide approximately 4 × 10^2^ PFU VV_IHD−J_/ml in the assays. The infection was performed in duplicate on confluent Vero cells, using 200 µl/Petri dish (5-cm diameter) and was allowed to proceed for 1 h at 37 °C. The same amount of virus incubated with DMEM was used as the control. Four days later, 1.8% neutral red was added, and the plaques were counted the next day, as described previously [28]. Serum samples from each experimental group were pooled for each timepoint. The neutralizing activity was calculated as the percent reduction in plaque number compared to the control, in which the virus was incubated with DMEM only.

### Statistical analyses

Statistical analyses were performed using one-way ANOVA parametric tests conducted with all groups at once and Bonferroni analysis of variance, using the GraphPad Prism 5 software. Statistical significance was set as *p* < 0.05 (*), *p* < 0.01 (**) and *p* < 0.001 (***).

## Results

### Specific humoral immune response is elicited in mice

**Fig. 2:**
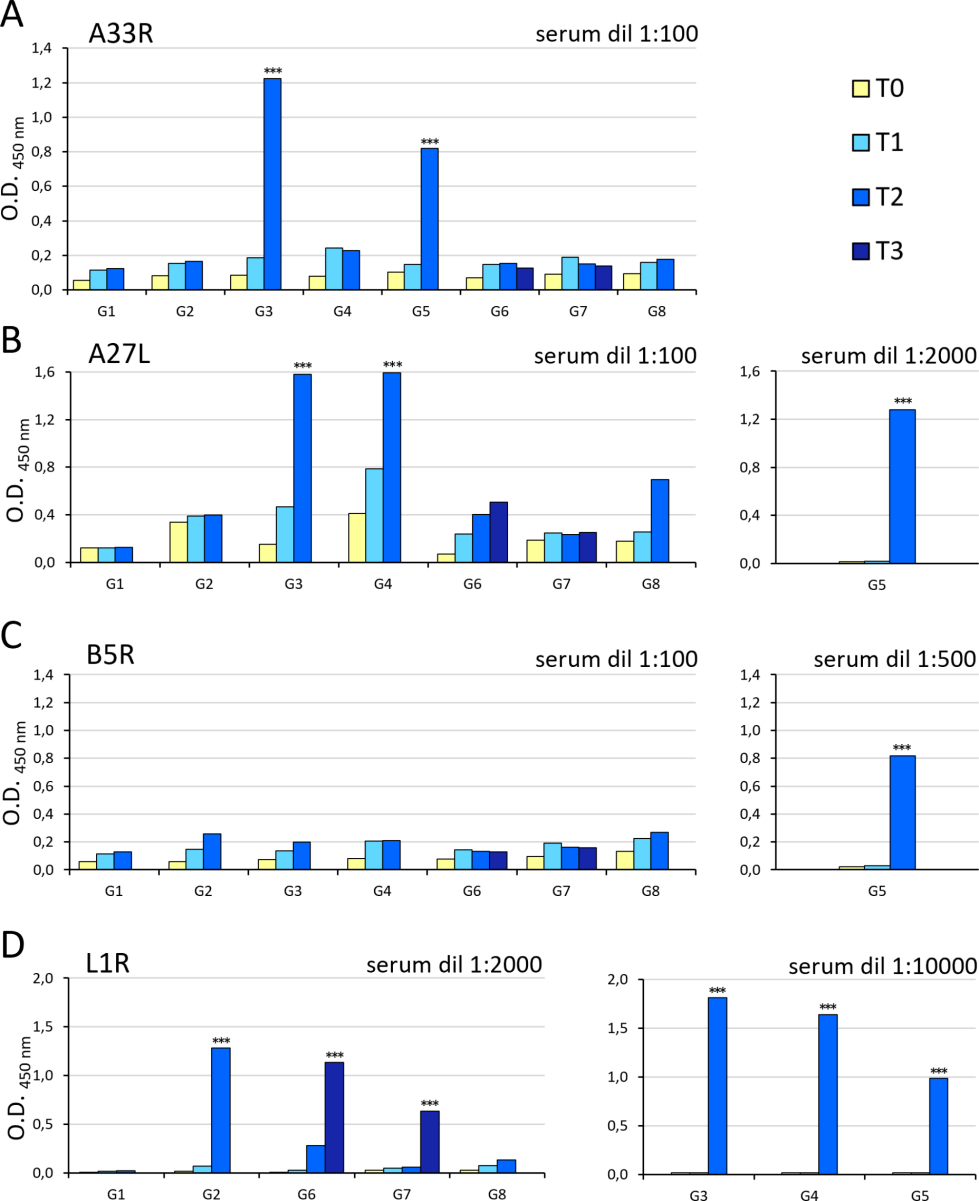
**Analysis of specific humoral responses by ELISA.** The sera of the mice from the eight groups were examined at the different times post immunization, using the individual proteins as plate-bound antigens. (A) when using the A33 protein, the antibody response, with a 1:100 serum dilution, was significantly greater for G3 and G5 (G3 and G5 vs. G1; *p* < 0.001); (B) for the A27 protein, the response, with 1:100 and 1:2000 serum dilutions, was significantly higher for G3, G4, G5 (G3, G4, G5 vs. G1; *p* < 0.001); (C) for the B5 protein, the response, with 1:100 and 1:500 serum dilutions, was significantly higher only for G5 (G5 vs. G1; *p* < 0.001); (D) for the L1 protein, the response, with 1:2000 and 1:10000 serum dilutions, was significantly higher for all of the groups (*p* < 0.001) except for G8. Statistical differences are shown (one-way ANOVA parametric tests, Bonferroni analysis of variance): ***, *p* < 0.001

With the aim to develop a protective vaccination strategy against OPXV infections, eight different immunization protocols were compared using a *prime–boost* strategy. The protocols were set up to verify their capability to induce antibodies against the VACV L1, A27, A33, and B5 proteins expressed by DNA or FWPV or recombinant proteins administered in combination. A new recombinant expressing the L1 protein was used, where the *L1R* open reading frame was modified with the tPA signal sequence to allow efficient expression of the protein on the cell surface. The specific humoral responses were measured by ELISA, using pooled sera from each group of immunized mice from blood drawn before and during the immunization protocols. A preliminary study with the pooled VACV proteins showed significant increases in humoral responses after the last *boost*, i.e. before the challenge, at a 1:5000 serum dilution (data not shown). Thus, we plated the individual A33, A27, B5, and L1 proteins to test the specificity of the antibodies that were induced by the vaccinations (Fig. 2A, B, C and D), for each antigen. All of the groups showed humoral responses against the single proteins with progressively higher values against B5, A33, A27, L1, respectively. After the last immunization, G5 showed significantly higher humoral responses against A33 (Fig. 2A; G5 vs. G1; *p* < 0.001), A27 (Fig. 2b; G5 vs. G1 and vs. G3 and G4; *p* < 0.001), B5 (Fig. 2C; G5 vs. all of the groups G1, G2, G3, G4, G6, G7, G8; *p* < 0.001), and the L1 proteins (Fig. 2D; G5 vs. G1, and G5 vs. G2, G6, G7; *p* < 0.001). Conversely, G2, G6, and G7 showed a significantly higher immune response against L1 at 1:2000 dilution compared to the G1 control group (Fig. 2D; G2, G6, G7 vs. G1; *p* < 0.001). However, the most significant increases in antibody responses were observed against L1 by G3 and G4 and G5 at 1:10000 dilution, when compared to the control and to all the other groups (G3, G4, G5 vs. all the groups; *p* < 0.001). Here, G5 did not reach the level attained by G3 and G4 (G3 and G4 vs. G5; *p* < 0.001). Also, the antibody response against A33 was significantly higher in G3 compared to G5 (Fig. A; G3 vs. G5; *p* < 0.001).

### Neutralizing activity against VV_IHD−J_ correlates with recovery from weight loss

**Fig. 3:**
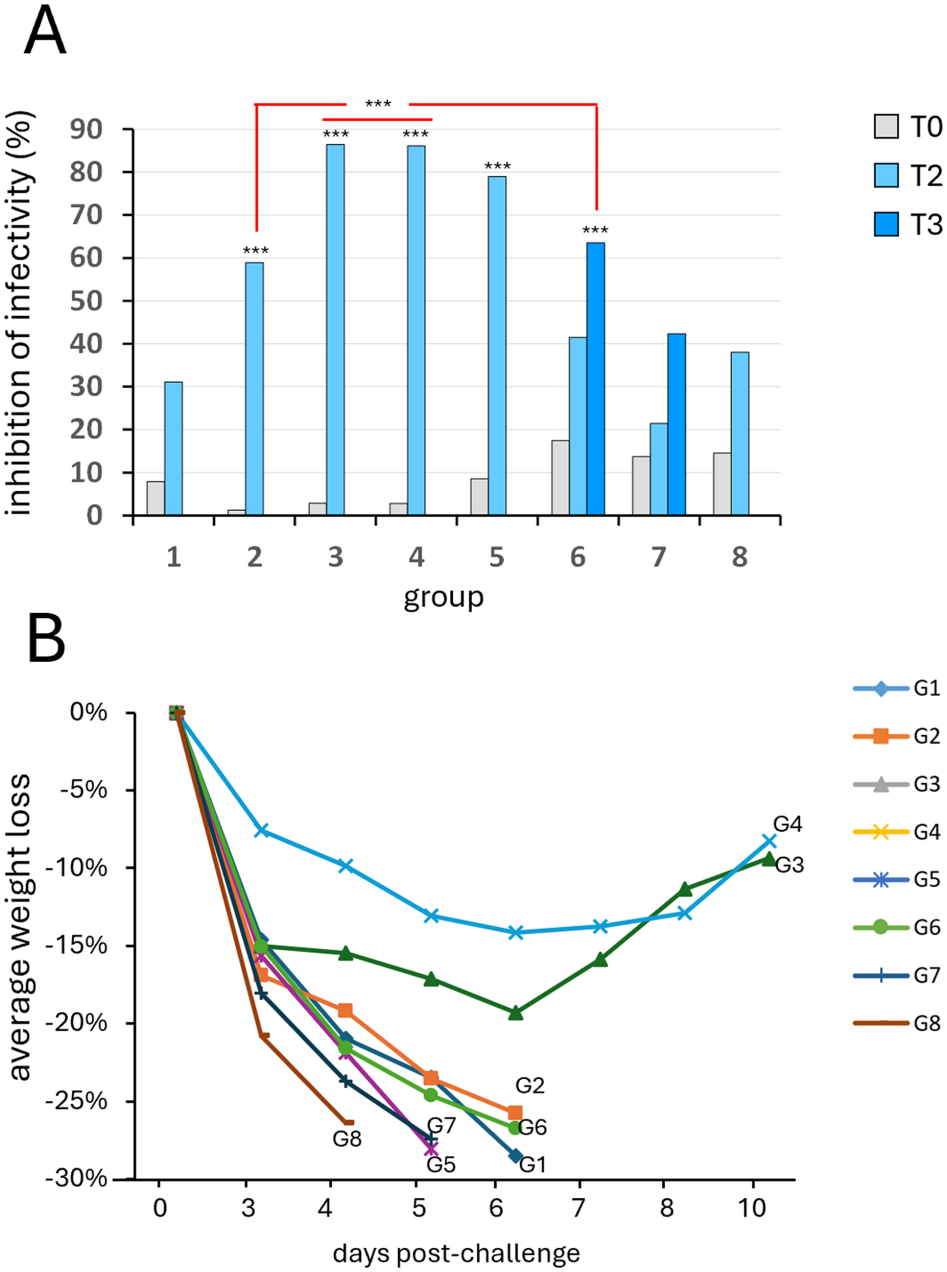
**Inhibition of viral infectivity by the different immunization protocols and effects on weight loss induced by the different immunization regimens after VV**_**IHD−J**_**challenge.** Viral plaque reduction neutralization tests (PRNT) were performed on Vero cells using sera before the first immunization (T0) and just before the challenge (T2 or T3). Plaque reduction was quantified, and expressed as percentages of inhibition of infectivity. (A) Using pooled sera from each group of mice, G2, G3, G4, G5, G6 showed significant inhibition of infectivity when compared with G1 (G2, G3, G4, G5 vs. G1, *p* < 0.001). Neutralization activity was significantly higher in G3 and G4 compared to G2 and G6 (G3 and G4 vs. G2 and G6; *p* < 0.001, internal comparisons). (B) All of the mice challenged with VV_IHD−J_ were monitored daily for the post-challenge (p.c.) percentage weight loss. Data are means of percentage weight loss of each group. Mice rapidly lost weight by day 3 p.c., as 15–22% with no relevant differences among most of the groups, but G3 and G4 mice started to regain weight after day 6 p.c. All of the G3 and G4 mice were protected and survived. G1 mice and mice of the remaining groups had to be euthanized from day 4 to 6 p.c. when their weight diminished by 25–30%, which represented the humane endpoint. Statistical differences are shown (one-way ANOVA parametric tests, Bonferroni analysis of variance): *, *p* < 0.05; **, *p* < 0.01; ***, *p* < 0.001; internal comparisons are indicated in red

To determine putative pre-challenge immune correlates of protection against VV_IHD−J_, viral plaque reduction tests (PRNTs) were performed using the sera from the different timepoints. These included pooled sera from the mice of each experimental group starting from the pre-immune serum (Fig. 3A; T0) up to the pre-challenge blood draw (T2 or T3). Results were assessed after subtraction of the pre-immune serum values. Inhibition of viral infectivity increased significantly in most of the groups after the last immunization, except for G7 and G8 (Fig. 3A; G2, G3, G4, G5, G6 vs. G1; *p* < 0.001). Neutralization activity was significantly higher in G3 and G4 than in G2 and G6 (Fig. 3A; G3 and G4 vs. G2 and G6; *p* < 0.001, internal comparisons). For all of the groups, no significant inhibition of viral infectivity was detected after the first immunization.

The weight of the mice, which was monitored daily, showed a rapid decrease after challenge in all of the animals at day 3 and 4 p.c. (Fig. 3B). This decrease was much less pronounced in the G3 and G4 animals, which regained their weight starting from day 6 p.c. This seems to correlate with the higher neutralizing activity of these two groups of mice, which were immunized with the same antigens (twice with 4pVAXmix plus twice with 4FPmix recombinants) administered by the i.n. and i.m. routes, respectively. It is noteworthy that none of the animals in G3 and G4 lost more than 25% of their weight, which represented the endpoint for euthanasia.

## Discussion

Several studies have demonstrated that combined systemic and mucosal *prime-boost* immunizations can enhance both the humoral and cellular arms of the immune response [54, 55]. The critical role of the humoral response against OPXVs has already been described [56, 57], and passive transfer of VACV-specific sera was shown to confer protection in both mice and monkeys [58]. In this context, vaccinations in which DNA *priming* was followed by recombinant viral vaccine *boosts* could elicit greater immunity when compared to the use of single immunogens [59–62]. Vaccine efficacy may also depend on inoculation site and recruitment of antigen presenting cells [54, 63]. Vaccination via i.m. and s.c. routes leads to stimulation of systemic immune responses, but is not efficient in promoting immune protection at mucosal membranes [64]. Conversely, i.n. mucosal immunization can trigger humoral and cell-mediated immunity both at mucosal sites and systemically [65, 66]. The development of live-attenuated or inactivated mucosal vaccines should therefore meet the needs for better protection against pathogens that penetrate through mucosal membranes [67]. The presence of high levels of IgAs in nasal lymphoid tissue and in the lungs, which are the respiratory pathways through which OPVXs infect animals and humans, can be fundamental for inhibition of viral attachment and penetration through the mucosal epithelium [68].

In the present study, mixtures of plasmids or FWPV recombinants expressing VACV *L1R*,* A27L*,* A33R*, and *B5R* genes as well as the corresponding recombinant proteins were administered following heterologous *prime-boost* immunization regimens using different routes, that is e.p./s.c. for DNA, i.n./i.m. for FWPV recombinants, and s.c. for recombinant proteins. Our aim was to compare different vaccination protocols and evaluate the humoral response, as well as protection for mice challenged by i.n. administration of the highly pathogenic VV_IHD−J_ strain.

Results demonstrated that: (i) all of the mice were protected against VV_IHD−J_ challenge when primed twice with 4pVAXmix and boosted twice with 4FPmix either i.n. or i.m.; (ii) protection directly correlated with a higher level of VV_IHD−J_ neutralizing antibodies and inversely correlated with weight loss; (iii) two protein *boosts* significantly enhanced the humoral response when following two *primes* with 4pVAXmix; (iv) single-shot *prime-boosts* with any combined immunogen were unable to elicit a significant humoral response; (v) the putative protective role of humoral immune responses appeared to be ascribed jointly to all of the different proteins, but mainly to L1, the expression of which was enhanced by the addition of the tPA sequence to the *L1R* amino terminus.

It is also interesting to note that: (1) Two doses of an irrelevant DNA (pVAX*gp*) appeared to prime by itself a humoral response but only against L1, and a neutralizing response almost as significant as the one elicited by the relevant plasmid DNA (G2 vs. G3); (2) protein *boosts* appeared to work almost as well as FWPV *boosts* in eliciting a humoral and neutralizing response, but recombinant proteins induced no protection (G3 vs. G5); (3) single-shots for each immunogen slightly increased the humoral and neutralizing responses, but only when *boosting* with the recombinant protein mixture and thanks to the L1 protein (G6 vs. G8); (4) *priming* with irrelevant DNA enhanced the Ab response against L1, but did not increase the neutralization response (G7 vs. G6).

In this study, the magnitude of the antibody response was variable in the different groups of animals and against the different proteins, and a significant increase was shown in G3, G4, G5 after the last *boost* immunization. No substantial enhancement of the antibody response was obtained when the immunogens were administered only once or when single DNA immunization was combined with single viral or protein recombinants (G6, G7, G8). In contrast to previous observations reported for MPXV orthologues [69], here the VACV L1 antigen was the most immunogenic, whereas a lower response was detected for B5, A33, and A27. In a previous paper [37], unmodified L1 protein was not recognized by any specific antibody, because of its very low expression on infected cells. We thus set out to increase its expression by constructing a novel fowlpox-based recombinant (FP_*tPA−L1R*_), in which the tissue plasminogen activator signal sequence (tPA) was added to the 5’ end of *L1R* open reading frame to drive the protein into the cellular secretion pathway. This L1 protein showed long-lasting expression in both Vero and MRC-5 mammalian cells that may have significantly enhanced the immunogenicity of this putative vaccine [38].

The protective efficacy of DNA immunization was demonstrated previously both in mice and non-human primates [40, 70, 71], but, in our hands, no significant response was shown either by *priming* with the DNA recombinants twice followed by irrelevant FWPV boosts [37] or by *priming* only once followed by *boosting* with the viral and protein recombinants (Fig. 1; G6). As a general drawback, DNA vaccines may show lower efficacy in non-human and human primates [72]. Their immunogenicity is generally enhanced when used as a *prime* followed by *boosts* with poxvirus-based recombinants, but various strategies are also being developed to address their lack of potency, including improvements in delivery methods. Electroporation creates transient increases in cell membrane permeability, and enhances DNA uptake, thus leading to a more robust immune response. This powerful technology is safe and well tolerated by patients, and improves immunological responses and vaccine efficacy [73, 74]. DNA vaccine genes can also be easily replaced to generate new recombinant immunogens, which may be useful for mass vaccination and can give a quick response to a new pandemic. Recently, most clinical studies of DNA-based vaccines for SARS-CoV-2 used electroporation (ClinicalTrials.gov), suggesting that electric transfer to deliver DNA is still the main trend. Therapeutic DNA vaccines against HPV-associated lesions/tumors are also based on electroporation as a delivery technique - VGX-3100, a DNA vaccine targeting HPV E6 and E7, is in a Phase-III clinical trial, which indicates its potential for licensing in the near future [75].

Neutralization of infectivity generally correlates with the level of antibodies against the viral surface antigens, and is usually a direct indication of vaccine efficacy and protection. In the present study, a basal neutralization was found in G1 against VACV, which was also previously described in the absence of any protection [37]. In contrast, virus-neutralizing antibodies increased significantly after the *boost* immunizations in G2, G3, G4, G5, and G6. However, an inverse correlation between neutralization and weight loss was mainly evident only in G3 and G4, where all of the animals regained weight from day 6 post challenge (p.c.). Animals in G2, G5 and G6 showed significant increases in neutralizing titers compared to G1, but lost more weight through day 6 p.c. and had to be sacrificed. A significant neutralizing activity was also present in G2 although the DNA immunogen used for *priming* was irrelevant. A partial contribution to immunogenicity by irrelevant DNA immunogens was also shown previously [37], which underlines the importance of inducing a higher neutralizing response (G3 vs. G2).

Previous studies indicated that mucosal immunization induces better protective efficacy, compared to systemic vaccination [76, 77]. In our in vivo experiments, G3 and G4 animals were immunized with the same immunogens given by i.n. or i.m. administration route, respectively, and the two groups showed a similar neutralization activity and a similar trend in recovering weight loss. It is likely that the double immunization with the relevant immunogens may be a prerequisite for protection. Although the recombinant proteins could induce neutralizing activity that was similar to that produced by 4 FWPV viral recombinants (G5 vs. G3) when used after the DNA immunogens, their efficacy was lower, as shown by the G5 animals, which could not recover their weight. Overall, neutralization was inversely correlated with weight decrease, which was initially similar in all of the groups after the VV_IHD−J_ challenge, but was then reversed in mice primed with the DNA recombinants and boosted with the FWPV recombinants.

## Conclusions

We previously demonstrated that DNA and FWPV recombinant vaccines expressing the VACV L1, A27, A33, and B5 proteins correlated with serum neutralizing activity and could protect mice against the highly pathogenic VV_IHD−J_. The administration of DNA recombinants by e.p. followed by FWPV recombinants by the i.n. immunization route seemed to be the key for protection [37]. However, in the present study, comparable protection was obtained by the i.m. and i.n administration routes using the same viral recombinants as immunogens. It is likely that the same DNA recombinants used for *priming*, boosted by FWPV recombinants, failed to protect previously [49] because only a single DNA *priming* step was performed. The double-shot *priming* with the same DNA recombinants in this study seems therefore to be critical for protection rather than FWPV administration via the i.m. or i.n. route. With this study, we can assess the failure of the one-shot immunizations also when using all of the three different immunogens. Moreover, recombinant proteins, even when administered twice, can boost the humoral response after DNA immunization, but cannot protect from viral challenge. Despite the limitation of the present study, these preliminary results provide information that will be useful for further investigations on the presence of a cell-mediated response, and the cross-immunity against other OPVXs, and enlarge the putative efficacy of the designed vaccine regimens. It is also evident that *prime* and *boost* immunizations with the same immunogens have to be repeated at least twice. In addition, the encouraging results obtained using L1R modified with the tPA leader sequence suggest that this approach may be useful to enhance secretion and immune recognition of other proteins.

## Data Availability

No datasets were generated or analysed during the current study.

## Declarations

### Declarations of Animal approval

The animals were maintained according to the Italian National Guidelines and the EU Directive 2010/63/EU for animal experiments. Approval for this study was granted by the Ethical Committee of the University of Milan and by the Italian Health Ministry (authorization n° 325/2020-PR of 14/04/2020).

## Abbreviations

CEFs: Chicken embryo fibroblasts
DMEM: Dulbecco’s Modified Eagle’s Medium
FWPV: Fowlpox virus
